# Supplementary material for: Evaluation of the Antibacterial Effect of Aurone-Derived Triazoles on Staphylococcus aureus
Source: Antibiotics (Basel). 2023 Aug 26;12(9):1370. doi: 10.3390/antibiotics12091370 (PMC10525585; doi:10.3390/antibiotics12091370)
Supplement: Supplementary file 1 [file antibiotics-12-01370-s001.zip › antibiotics-2562984-supplementary/TableS1-AT Compounds.pdf]

Table S1: Anti-staphylococcal activity of aurone-triazole (AT) compounds. A total of 25 aurone-triazole (AT) compounds at 100 µM concentrations were tested for anti-Staphylococcal activity. AT compounds with > 60% inhibition against either methicillin-sensitive *Staphylococcus aureus* (MSSA; *S. aureus* ATCC strain 29213) or methicillin-resistant *S. aureus* (MRSA; *S. aureus* ATCC 43300) were chosen for further testing to determine the IC_{50} (50% *S. aureus* inhibitory concentration) and CC_{50} values (50% human cell inhibitory concentration) for human HepG2 liver cells and/or HeLa cells. The selectivity index (SI) for mammalian cell toxicity of the ATs was calculated as CC_{50}:IC_{50}. An SI > 10 indicates the compound has selectivity towards the bacterium and not the human cells. The table shows the SI ratio of HepG2 cells to either MSSA or MRSA (best ratio). The (-) symbol indicates that the test was not done. Compounds marked with (*) were the focus of this study.

Sample ID	Compound	MSSA inhibition (100µM)	MRSA inhibition (100µM)	IC_{50} MSSA (µM)	IC_{50} MRSA (µM)	CC_{50} HepG2 (µM)	CC_{50} HeLa (µM)	SI
**AT100**	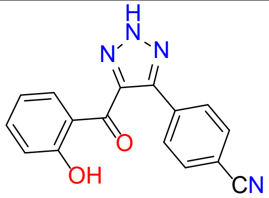	58.77%	96.47%	-	38.84	70.66	19.07	1.82
**AT102**	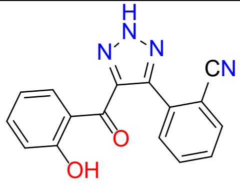	0%	35.77%	-	-	-	-	-
**AT103**	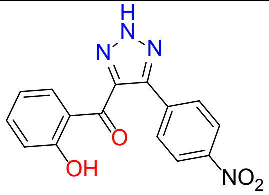	81.57%	88.53%	-	3.983	2.65	-	0.67
**AT104**	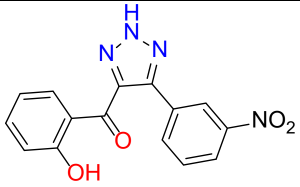	-	48.19%	-	-	-	-	-
* **AT106**	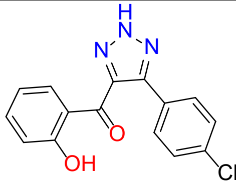	87.39%	64.13%	5.439	23.54	70.66	16.19	12.99

Sample ID	Compound	MSSA inhibition (100µM)	MRSA inhibition (100µM)	IC_{50} MSSA (µM)	IC_{50} MRSA (µM)	CC_{50} HepG2 (µM)	CC_{50} HeLa (µM)	SI
**AT107**	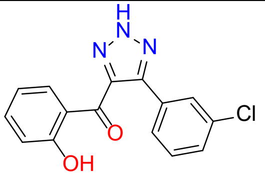	50.24%	56.47%	-	-	-	-	-
**AT108**	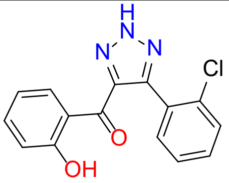	30.63%	8.46%	-	-	-	-	-
**AT113**	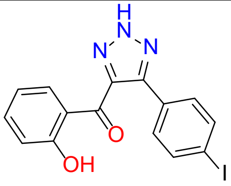	85.79%	43.81%	-	-	-	-	-
* **AT116**	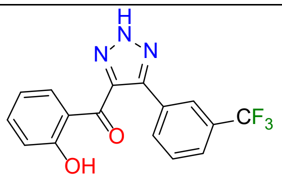	81.95%	100%	3.178	35.38	50.87	11.7	16.01
**AT117**	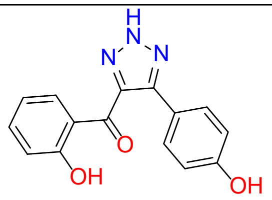	0%	19.39%	-	-	-	-	-
**AT119**	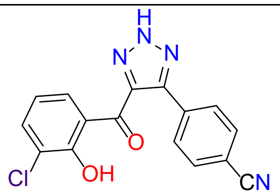	64.36%	46.21%	-	-	-	-	-
**AT120**	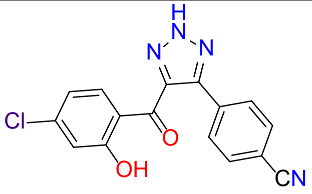	71.82%	99.51%	6.025	4.182	38.12	17.15	9.12

Sample ID	Compound	MSSA inhibition (100µM)	MRSA inhibition (100µM)	IC_{50} MSSA (µM)	IC_{50} MRSA (µM)	CC_{50} HepG2 (µM)	CC_{50} HeLa (µM)	SI
**AT122**	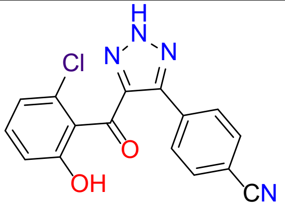	1.01%	18.51%	-	-	-	-	-
**AT123**	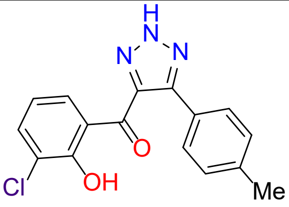	99.45%	23.62%	12.46	31.16	58.68	14.4	4.71
**AT124**	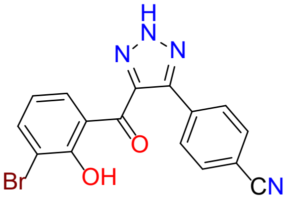	43.36%	32.16%	-	13.36	3.851	-	0.29
* **AT125**	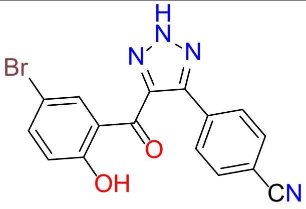	85.00%	60.47%	4.325	5.412	50.57	9.073	11.69
**AT126**	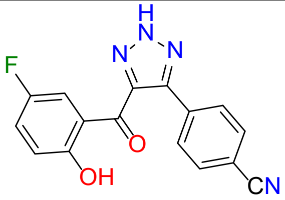	64.72%	75.06%	-	-	-	-	-
**AT130**	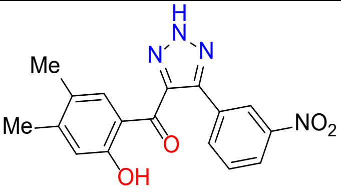	58.75%	43.97%	-	-	-	-	-

Sample ID	Compound	MSSA inhibition (100µM)	MRSA inhibition (100µM)	IC_{50} MSSA (µM)	IC_{50} MRSA (µM)	CC_{50} HepG2 (µM)	CC_{50} HeLa (µM)	SI
**AT131**	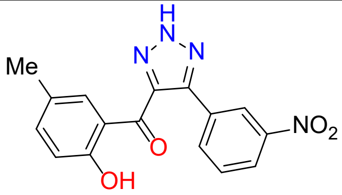	21.31%	0%	-	-	-	-	-
**AT132**	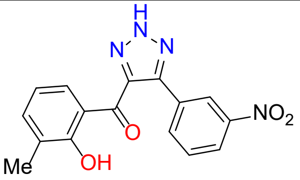	83.03%	57.99%	-	-	-	-	-
**AT133**	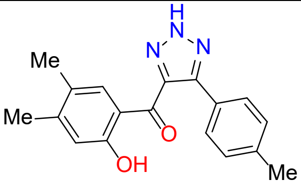	88.47%	58.84%	-	-	-	-	-
**AT134**	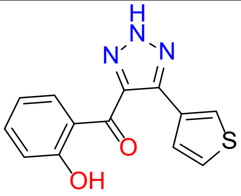	10.83%	0%	-	-	-	-	-
**AT135**	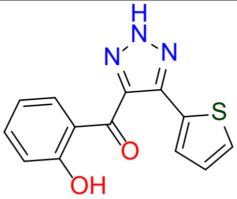	46.76%	5.42%	-	-	-	-	-
**AT136**	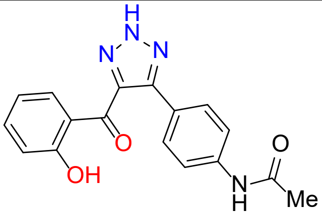	0%	0%	-	-	-	-	-
* **AT137**	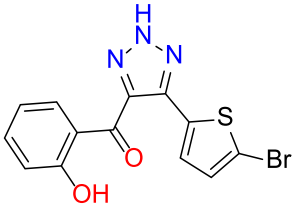	87.46%	93.84%	3.092	3.87	39.81	12.67	12.86
